# Pharmacological insights into *Merremia vitifolia* (Burm.f.) Hallier f. leaf for its antioxidant, thrombolytic, anti-arthritic and anti-nociceptive potential

**DOI:** 10.1042/BSR20203022

**Published:** 2021-01-07

**Authors:** Sahida Akter, Israt Jahan, Mst. Riniara Khatun, Mohammad Forhad Khan, Laiba Arshad, Md. Jakaria, Md. Areeful Haque

**Affiliations:** 1 Department of Pharmacy, International Islamic University Chittagong, Chittagong 4318, Bangladesh; 2 Department of Pharmacy, Forman Christian College (A Chartered University), Lahore, Pakistan; 3 Melbourne Dementia Research Centre, The Florey Institute of Neuroscience and Mental Health, The University of Melbourne, Parkville, VIC 3052, Australia; 4 Drug & Herbal Research Centre, Faculty of Pharmacy, Universiti Kebangsaan Malaysia, 50300 Kuala Lumpur, Malaysia

**Keywords:** Merremia vitifolia, anti-arthritic, anti-nociceptive activities, antioxidant, DPPH, thrombolytic

## Abstract

*Merremia vitifolia* (Burm.f.) Hallier f., an ethnomedicinally important plant, used in the tribal areas to treat various ailments including fever, headache, eye inflammation, rheumatism, dysentery, jaundice and urinary diseases. The present study explored the biological efficacy of the aqueous fraction of *M. vitifolia* leaves (AFMV) through *in vitro* and *in vivo* experimental models. The thrombolytic and anti-arthritic effects of AFMV were evaluated by using the clot lysis technique and inhibition of protein denaturation technique, respectively. The anti-nociceptive activity of AFMV was investigated in Swiss Albino mice by acetic acid-induced writhing test and formalin-induced paw licking test. The antioxidant activities of AFMV, including 2,2-diphenyl-1-picrylhydrazyl (DPPH) free radical and total reducing power, were also tested. The qualitative phytochemical assays exhibited AFMV contains secondary metabolites such as alkaloid, carbohydrate, flavonoid, tannin, triterpenoids and phenols. In addition, AFMV showed strong antioxidant effects with the highest scavenging activity (IC_50_ 146.61 µg/mL) and reducing power was increased with a dose-dependent manner. AFMV also revealed notable clot lysis effect and substantial anti-arthritic activity at higher doses (500 µg/mL) as compared with the control. The results demonstrated a promising reduction of the number of writhing and duration of paw licking in acetic acid-induced writhing test and formalin-induced paw licking test in a dose-dependent manner, respectively. In conclusion, AFMV provides the scientific basis of its folkloric usage, suggesting it as the vital source of dietary supplement.

## Introduction

Natural product is known as a healthy source of life for all people, and plants and plant-based dietary supplements have been used due to their wide range of medicinal and nutritional values [1]. From prehistoric ages, various natural productsand plant-derived supplement were used by all societies and civilisations for the management of diverse diseases [2]. According to the World Health Organization, 80% of the world’s population relies on herbal or traditional medications owing to the safety and economic reasons [3].

Oxidative stress is associated with the pathogenesis of several chronic diseases and disorders such as cardiac diseases, neurodegenerative diseases, diabetes and cancer [4], and plant-based secondary metabolites play protective role against reactive oxygen species (ROS) mediated disorders [5–8]. However, the identification of natural antioxidants and their source has drawn attention over synthetic drugs owing to their benefits. Rheumatoid arthritis (RA) is a chronic inflammatory autoimmune disorder associated with genetic pathophysiology and environmental factors primarily responsible for the destruction of synovial fluids, inflammatory cartilage degeneration, morning stiffness, bone alcoholysis and joint deformities [9]. Recent research studies revealed that approximately 0.5–1% of people are globally suffered from rheumatoid arthritis, where women are affected more than men, women: men (3:1) [10]. Notably, ROS, lipoxygenase, pro-inflammatory cytokines (such as tumour necrosis factor-α, interleukin (IL)-1β), prostaglandin-endoperoxide synthase, prostaglandins, cyclooxygenase-2 (COX-2) enzyme and macrophage colony-stimulating factor plays a crucial role in the pathophysiology of rheumatoid arthritis. In the rheumatoid synovium, the activation of polymorphonuclear cells and macrophages enhanced O_2_ consumption with the release of toxic free radicals, which can damage joint and cartilage tissue that leads to triggers an inflammatory response and cytolysis [11]. This process can reduce antioxidant defence system (glutathione, superoxide dismutase and catalase) or increase in lipid peroxidation marker (such as malondialdehyde).

Thrombosis is a lethal disease, which causes acute coronary disorders, for example, (ischemia, coronary artery diseases, myocardial infarction and atherosclerosis) that accounts for sudden morbidity or mortality [12]. Blood clots or thrombus formed in the circulatory system due to homeostatic imbalance between thrombogenic factors and protective mechanisms, causing vascular blockage and depriving tissues normal flow of blood and oxygen, this causes ischemia of the tissues throughout the region and leads to fatal consequences ultimately prompting death [13]. Numerous thrombolytic agents are used to treat myocardial infarction; among them, streptokinase (SK) is notable but leads to an increased risk of severe hemorrhagic transformation, anaphylactic reaction and hypertension. Treatment with the alternative option of medicines such as traditional and herbal drugs is frequently being searched for their safety profile to overcome current adverse effects of this therapy based on the high importance of the thrombolytic agents.

*Merremia vitifolia* (Burm. f.) Hallier f. is a perennial, climbing plant with twining stems can be 2–5 meters long and widely distributed in Bangladesh, India, Srilanka, Myanmar, Thailand and throughout Malaysia. Traditionally, leaf and rhizome of this plant are used in tribal areas to treat numerous illness such as fever, headache, eye inflammation, rheumatism, dysentery, jaundice and urinary diseases [14]. Considering the enormous ethnomedicinal uses and therapeutic value of *M. vitifolia*, this research was aimed to elucidate the *in vitro* antioxidative potential, thrombolytic effect, anti-arthritic action and *in vivo* anti-nociceptive effect of aqueous extract of *M. vitifolia* leaves (AFMV) to determine its relevance to treating certain disorders.

## Materials and methods

### Chemicals and reagents

Methanol, acetic acid, *n*-hexane, ethyl acetate, Folin-Ciocalteu reagent, potassium ferricyanide from Sigma Chemical Company, St. Louis, MO, U.S.A.); lyophilised SK vial (1500000 IU) and diclofenac sodium (standard drugs) were procured from the Incepta Pharmaceuticals Ltd., Bangladesh. Normal saline solution (0.9% NaCl) was obtained from the Social Marketing Company (SMC) Ltd, Bangladesh, and Tween-80 was patronised from BDH Chemicals (Leicestershire, U.K.). All other chemicals of analytical grade were purchased from local sources through Taj Scientific Ltd., Bangladesh.

### Animals

Animals were maintained as the published article by Siddiqui et al. (2020) [15]. Six to 7 week-old Swiss albino mice weighing 22–30 g were purchased from the animal research division of the Jahangirnagar University, Dhaka, Bangladesh. The mice were maintained at room temperature (23 ± 2°C) and 55–65% humidity with a natural 12:12-h light and dark cycle. All the mouse was acclimatised to a new environment for 7 days preceding to the experiment. The mouse was supplied with standard laboratory diet *ad libitum* and freshwater, and mouse fasted overnight before the experiment. The experiment was approved by the Animal Ethical Committee, Department of Pharmacy, protocol number: 2019/65, International Islamic University Chittagong, Bangladesh. All experiments were done at the Department of Pharmacy, International Islamic University Chittagong, Bangladesh. The animal study was observational or behavioural, and no surgery or scarification was implicated for this experiment.

### Acute toxicity test

Mice were healthy at the time of acclimatisation in the laboratory environment before the commencement of the toxicity analysis. The study was conducted according to the OECD guidelines and following the described method with little modification [16,17]. Male Swiss albino mice were divided into seven groups, consisting of six animals (n=6) each. Test groups were administered different doses (200, 400, 800, 1600, 3000 and 4000 mg/kg, p.o.) while the control group received 1% tween 80 in saline water. The animals were monitored closely for the next 48 h and up to 14 consecutive days to record any acute toxicological signs (mortality, allergic reaction and behavioural changes).

### Plant materials

Fresh leaves of *M. vitifolia* were collected randomly from hill tract area of Chittagong, Bangladesh. A taxonomist then verified the collected plant samples, and a voucher specimen has been deposited with a number (accession#75488) for future references. The extraneous, objectionable contaminants were removed by hand and then washed the plant materials using freshwater. Washed plant materials were dried in shaded place at low temperature (45–50°C) and crushed into coarse powered using a mechanical grinder.

### Plant extraction and fraction preparation

Approximately 500 g coarse powdered leaves were dissolved in 3000 mL of methanol at a ratio of 1:6 (plant sample: solvent) at room temperature (25 ± 2°C) for 3 days with occasional shaking and stirring on a shaker machine and then filtered using cotton followed by Whatman #1 filter paper. The filtrate was concentrated by evaporating the solvent using a water bath at a temperature of 40–45°C to yield a viscous mass of crude methanol extract. Around 50 g of the crude methanol extract was fractionated, followed by modified Kupchan method [18] (Kupchan et al., 1973). Approximately 8 g of ethyl acetate fraction and 14 g of aqueous fraction were obtained. The fractions were stored at 4°C in airtight containers, and the AFMV was dissolved in saline water with 1% tween 80 before the experiments.

### Phytochemical analysis

The AFMV extracts were subjected to a qualitative phytochemical analysis using standard procedures for assessing the existence of alkaloids, carbohydrates, glycosides, flavonoids, tannins, saponins, phenols and proteins [19,20].

### Antioxidant activity

#### Determination of DPPH free radical scavenging activity

The free radical scavenging activity of AFMV was assessed using stable DPPH (1,1-diphenyl-2-picrylhydrazil) according to the procedure mentioned earlier [16]. A 3 ml of 0.004% DPPH solution (4 mg DPPH in 100 ml 95% methanol) was dissolved in different concentration of either extract or standard (31.25–500 µg/ml). After 30 min incubation at 25°C, the absorbance was taken at 517 nm using UV-visible spectrophotometer (AE-S90, A & E Lab, U.K. CO., LTD.). The percentage (%) of inhibition was calculated using following the formula,

$$\left[\frac{{\text{A}}_{0}-{\text{A}}_{1}}{{\text{A}}_{0}}\right]\times 100$$

Where, *A*_0_ = control absorbance and *A*_1_ = sample/standard absorbance

#### Determination of reducing power capacity

The reducing power assay was investigated according to the modified method [16]. About 1 ml of the test sample or standard at different concentrations level (31.25–500 μg/mL) was mixed with 2.5 ml of phosphate buffer (0.2 M, pH 6.6) and potassium ferricyanide (1% w/v), respectively and incubated for 20 min at 50°C to complete the reaction. The mixture was then centrifuged (at 3000 rpm for 10 min) with added 2.5 ml of 10% trichloroacetic acid solution for put an end to the reaction. The supernatant (upper layer) solution was discarded, and 2.5 ml of distilled H_2_0 and 0.5 ml FeCl_3_ (1% w/v) were added, a visible Prussian blue colour indicating the endpoint of the reaction. Finally, the absorbance was taken at 700 nm by using UV-visible spectrophotometer (AE-S90, A & E Lab, U.K. CO., LTD.) against blank.

### Thrombolytic activity

The thrombolytic activity of AMFV was evaluated following the clot lysis test [21–23]. About 5 ml of venous blood drawn from ten healthy adult volunteers (Consent approval number: ECPD-IIUC-2019/02) 1:1 (male: female) with no history of an oral contraceptive and anticoagulant treatment. Consent was taken from the participants before collecting the blood sample. Blood from each volunteer distributed in nine pre-weighed sterile Eppendorf tube (0.5 ml/tube) and incubated for 45 min at 37°C to form the clot. After clot formation, serum was removed carefully without disrupting the clot, and each tube was reweighed to define the clot weight. In each tube having the pre-weighed clot, 100 µl of AFMV as the positive control, 100 µl of normal saline as negative control and 100 µl of SK as positive control were separately added. Incubation was done for 90 min at 37°C to notice clot lysis. After incubation, released fluid was separated using micropipette and tubes were reweighed to get the difference in clot weight after clot disruption. The percentage of clot lysis was calculated using the following formula:

$$\%\text{ of clot lysis}=\left(\frac{\text{weight of released clot}}{\text{clot weight}}\right)\times 100$$

### Anti-arthritic activity

The anti-arthritic activity of AFMV was evaluated using the inhibition of protein denaturation technique followed by a method described by Ansari et al. (2017) [24] with some modifications. Serially diluted concentrations between (31.25–500 µg/ml) of AFMV extract and standard diclofenac-Na were obtained. For each concentration 0.5 ml of test/standard solution from prepared dilutions was mixed with aqueous albumin (0.45 ml, 5% w/v) and 1N HCl was then added to adjust the (pH 6.3). The prepared mixtures then incubated at 37°C for 20 min after that the temperature was increased to keep the mixtures at 57°C for 30 min, the samples were cooled, and (2.5 ml) of phosphate buffer was added to the solution. All the reagents except extract are contained in the blank solution. The absorbance was measured using UV-Visible spectrophotometer at 416 nm. Each test was done for triplicate for maintaining accuracy. The % of inhibition of protein denaturation was calculated from the following equation:

$$\left[\frac{{\text{A}}_{0}-{\text{A}}_{1}}{{\text{A}}_{0}}\right]\times 100$$

Where *A*_0_ = control absorbance and *A*_1_ = sample/standard absorbance.

### Anti-nociceptive activity

#### Experimental design

In *in vivo* animal experiment, 24 animals were divided into four groups where each group consists of six animals (n=6). The standard drug diclofenac sodium (10 mg/kg, b.w, i.p.) was used for acetic acid-induced writhing test and formalin-induced paw licking test. The test groups were administrated AFMV at doses of 200 and 400 (mg/kg, b.w, p.o.), respectively, whereas the control group received vehicle (1% Tween 80 in saline water, 10 mL/kg, p.o). The standard drugs (diclofenac sodium) were administrated at 15 min and AFMV (200 and 400 mg/kg, p.o.) or vehicle at 30 min before the experiments.

#### Acetic acid-induced writhing test

The acetic acid-induced writhing test in swiss albino mice was carried out as described with minor modification [25–27]. All the animals received respective doses 30 min before initiating the study as per the experimental design. One hour later, mice in all groups were treated with 0.7% (v/v) acetic acid solution (10 mL/kg body weight, i.p). Five minutes after acetic acid injection (i.p), mice were placed in a glass made cylindrical cage, and the number of writhing and stretching was counted over 20 min. The percent inhibition of writhing was calculated from [*M*_c_ − *M*_t_/*M*_c_] × 100; where *M*_c_ = mean control and *M*_t_ = mean test. The overall study was observational or behavioural, and no surgery or scarification was implicated for this experiment.

#### Formalin-induced paw licking test

The formalin-induced paw licking test in Swiss albino mice was evaluated as previously described method [25,26]. All the animals were received with respective treatment 30 min before the commencement of the study as described in the experimental design section. After 30 min of the administration, (2.5%, 20 µL) formalin was injected by a micro-syringe into the sub-plantar region of the right hind paw of mice and placed in a glass made a cage for observation. The licking and biting of the injected paw were pain response and the first 5 min as neurogenic phase and later 15–30 min as the inflammatory phase was recorded. The % of inhibition of licking was calculated from [*M*_c_ − *M*_t_/*M*_c_] × 100, where *M*_c_ = mean control and *M*_t_ = mean test. The overall study was observational or behavioural, and no surgery or scarification was implicated for this experiment.

### Statistical analysis

The data were represented as (MEAN ± SEM), and the method was carried out using the GraphPad Prism Version 8.0 (GraphPad Software Inc, San Diego, CA). The statistical variance was dictated by one-way variance of analysis (ANOVA) followed by Dunnett’s test, *t*-test (nonparametric test) and two-way variances of analysis (ANOVA). Values of *P* is less than 0.05 and 0.01 considered as statistically significant and 0*.*001 were regarded as highly significant.

## Results

### Acute toxicity test

The oral administration of AFMV doses up to 4000 mg/kg was found tolerable and safe doses in experimental animals during close monitoring. There were no significant changes in the body weight, behavioural changes, stool frequency, water and food intake irregularity of toxicity and mortality were noted during this investigation.

### Phytochemical screening

The qualitative phytochemical screening of AFMV showed alkaloid, carbohydrate, flavonoids, tannins, protein and phenolic compounds (Supplementary Table S1).

### Antioxidant activity

#### DPPH free radical scavenging activity

The result of DPPH activity of AFMV extract and ascorbic acid are presented in (Table 1). However, a dose-dependent radical scavenging effect was observed in the DPPH assay. Among all concentrations, AFMV showed the highest scavenging (*P<0.001*) activity (76.11 ± 1.05%) with IC_50_ of 146.61 µg/mL at the concentration of 500 µg/mL, while the ascorbic acid exhibited 84.62 ± 0.74% with IC50 30.38 µg/mL. The outcomes suggested that AFMV can decrease OS-induced free radicals.

**Table 1:** DPPH scavenging activity of AFMV and ascorbic acid at different concentrations

Test material	Concentration (µg/mL^−1^)	% Scavenging activity (Mean ± SEM)	IC_50_ (μg mL^−1^)
Ascorbic acid	31.25	39.86 ± 0.31*^b^*	
	62.50	51.74 ± 0.89*^b^*	
	125.00	65.39 ± 0.44*^b^*	30.38
	250.00	74.56 ± 0.54*^c^*	
	500.00	84.62 ± 0.74*^c^*	
AFMV	31.25	27.30 ± 0.71*^a^*	
	62.50	39.29 ± 0.77*^b^*	
	125.00	50.31 ± 0.42*^b^*	146.61
	250.00	61.94 ± 1.02*^b^*	
	500.00	76.11 ± 1.05*^c^*	

All results are presented as mean ± SEM (n=3). Two-way analysis of variance (ANOVA) followed by Bonferroni post-test was employed where ***^a^****P<*0.05, ***^b^****P<*0.01, ***^c^****P*<0.001, were considered as statistically significant compared with the control; AFMV, aqueous fraction of *Merremia vitifolia* leaves.

#### Reducing power activity

As depicted in the (Table 2) AFMV extract showed substantial reducing activity (*P<0.05*) with the increasing of concentration-dependent manner as compared with ascorbic acid. The extract displayed higher reductive capability 1.113 ± 0.010, while the positive control ascorbic acid showed 2.189 ± 0.020 at the concentration of 500 µg/mL.

**Table 2:** Reducing power assay of AFMV at different concentration

Test material	Concentration (µg/mL^−1^)	Absorbance (Mean ± SEM)
Ascorbic acid	31.25	0.943 ± 0.030*^a^*
	62.50	1.278 ± 0.010*^a^*
	125.00	1.621 ± 0.030*^a^*
	250.00	1.828 ± 0.030*^a^*
	500.00	2.189 ± 0.020*^b^*
AFMV	31.25	0.520 ± 0.010*^a^*
	62.50	0.673 ± 0.020*^a^*
	125.00	0.867 ± 0.010*^a^*
	250.00	0.979 ± 0.010*^a^*
	500.00	1.113 ± 0.010*^b^*

Values are represented in Mean ± SEM (n=3). *t*-test (nonparametric test) was employed where ***^a^****P<*0.05, ***^b^****P<*0.01, ***^c^****P*<0.001 were considered as statistically significant compared to the reference drug; AFMV, aqueous fraction of *Merremia vitifolia* leaves.

### Thrombolytic activity

The clot lysis activity of the AFMV is presented in (Table 3). The AFMV revealed 42.48 ± 1.44% Clot lysis, while positive control (SK) showed 72.16 ± 1.85% of clot lysis. The AFMV demonstrated statistically significant (*P<0.001*) as compared with the negative control.

**Table 3:** The clot lysis activity of AFMV and streptokinase

Drug	% of clot lysis (Mean ± SEM)
Control (normal saline)	4.80 ± 2.45
Standard drug (streptokinase)	72.16 ± 1.85*^c^*
AFMV	42.48 ± 1.44*^c^*

All results are mean ± SEM (*n*=3). One-way analysis of variance (ANOVA) followed by Dunnett's test was employed where ***^a^****P<*0.05, ***^b^****P<*0.01, ***^c^****P*<0.001, were considered as statistically significant compared to the control; AFMV, aqueous fraction of *Merremia vitifolia* leaves.

### Anti-arthritic activity

In the present investigation, AFMV and diclofenac sodium (standard) exhibited significant (*P<0.05*) inhibition of protein denaturation in a dose-dependent manner, as shown in (Table 4). The extract showed the highest % of inhibition (63.87 ± 1.74%) at 500 µg/mL, wherein the standard drug (diclofenac sodium) showed 86.78 ± 1.92% at the same concentration.

**Table 4:** Effects of AFMV on protein denaturation compared to the standard drug diclofenac sodium

Diclofenac sodium		AFMV	
Concentration (µg/mL)	% of Inhibition (Mean ± SEM)	Concentration (µg/mL)	% of inhibition (Mean ± SEM)
31.25	51.29*^a^* ± 1.77	31.25	26.56*^a^* ± 1.57
62.5	58.95*^a^* ± 2.30	62.5	44.46*^a^* ± 1.42
125	70.57*^b^* ± 1.37	125	51.93*^a^* ± 2.03
250	81.19*^b^* ± 2.62	250	55.28*^a^* ± 2.10
500	86.78*^c^* ± 1.92	500	63.87*^b^* ± 1.74

Results are represented in mean ± SEM (n=3). Significance variance was followed by *t*-test, ***^a^****P<*0.05, ***^b^****P<*0.01, ***^c^****P*<0.001, were considered as statistically significant compared to the reference drug; AFMV, aqueous fraction of *Merremia vitifolia* leaves.

### Anti-nociceptive activity

#### Acetic acid-induced writhing test

The effects of the oral administration of the AFMV solution in the number of writhing on the acetic acid-induced test were shown in (Table 5). In the present study, AFMV (200 and 400 mg/kg, p.o.) and diclofenac sodium (10 mg/kg, i.p.) dose significantly (*P<0.001*) decreased the number of abdominal contortions by increasing the test doses (43.64 ± 0.60% and 29.92 ± 0.98%) and (21 ± 0.57%), respectively compared to the control group. However, the present outcomes indicating a dose-dependent effect.

**Table 5:** Effect of AFMV extract in acetic acid-writhing response in Swiss albino mice

Treatment	Dose	No. of writhes (Mean ± SEM)
Control (1% tween 80 in DW)	10 ml/kg, p.o.	56.45 ± 2.04
Standard (Diclofenac sodium)	10 mg/kg, i.p.	21 ± 0.57*^c^*
AFMV	200 mg/kg, p.o.	43.64 ± 0.60*^b^*
AFMV	400 mg/kg, p.o.	29.92 ± 0.98*^c^*

Values are represented in Mean ± SEM (n=6) and ^a^*P*<0.05, ^b^*P*<0.01, ^c^*P*<0.001 were considered as statistically significant compared to the control; AFMV, aqueous fraction of *Merremia vitifolia* leaves.

#### Formalin-induced paw licking assay

The effect of AFMV on formalin-induced nociception is presented in (Table 6). In the formalin-induced licking test, pain responses were evaluated individually in both early and late phases through analysing the duration of licking in animal models. The AFMV at 200 and 400 (mg/kg, p.o.) extract showed significant (*P<0.01*) and dose-dependent anti-nociceptive effect in both phases (early and late) of the test (44.19 ± 1.00% and 26.45 ± 1.05%) and (30.43 ± 1.02% and 19.89 ± 1.10%) though reducing paw licking duration in a meaningful manner when compared with the vehicle group. The reference drug diclofenac sodium significantly exerted 20.43 ± 0.73 and 15.16 ± 0.87% inhibition, at the same concentrations.

**Table 6:** Effect of AFMV extract in formalin induced-licking response in Swiss albino mice

Treatment	Dose	Duration of paw licking (S) (Mean ± SEM)	
		Early phase	Late phase
Control (1% tween 80 in DW)	10 ml/kg, p.o.	56.16 ± 1.0	37.54 ± 1.30
Standard (Diclofenac sodium)	10 mg/kg, i.p.	20.43 ± 0.73*^c^*	15.16 ± 0.87^c^
AFMV	200 mg/kg, p.o.	44.19 ± 1.0*^a^*	26.45 ± 1.05^b^
AFMV	400 mg/kg, p.o.	30.43 ± 1.02*^c^*	19.89 ± 0.1.10^c^

Values are represented in Mean ± SEM (n=6). One-way analysis of variance (ANOVA) followed by Dunnett’s test was employed where ***^a^****P<*0.05, ***^b^****P<*0.01, ***^c^****P*<0.001, were considered as statistically significant compared to the reference drug; AFMV, aqueous fraction of *Merremia vitifolia* leaves.

## Discussion

The modern discovery of natural products based on ethnomedicinal uses of the medicinal herb has been used for their therapeutic values since ancient times. Plant-derived bioactive compounds are commonly used in the pharmaceutical, nutritional and food supplement industries to prepare different herbal products, vitamins, food supplements and drugs for treating different disease [1]. The importance of medicinal plants in managing a wide range of diseases is progressively increasing because of their rich source of bioactive secondary metabolites [28–32]. Alkaloids, flavonoids, phenols, terpenoids and glycosides are important metabolites exhibit multiple biological effects [33,34]. Several well-validated experimental protocols and animal models are necessary for developing a pharmacologically complex lead compound derived from a medicinal plant to achieve a clear preclinical and clinical decision. Phytochemical analysis of the plant extract reveals several compounds considered responsible for numerous biological effects [35]. AFMV extracts demonstrated that the plant contains several secondary metabolites including alkaloid, carbohydrate, flavonoids, tannins, protein and phenolic. Alkaloids, common secondary nitrogenous compounds, used to treat various human and animal disorders since middle age and flavonoids are used to treat anti-inflammatory, cancer and cardiovascular diseases [36].

The homeostatic balance between free radicals and antioxidants play a central role in the expression of genes and receptor activation. However, a negative shift in the levels of free radicals can become harmful for the biological system, resulting in several diseases like inflammation, neurodegenerative disorders, aging and cancer [37]. Antioxidants counteract abnormal effects in the body associated with free radical damage and oxidative stress. Therefore, natural products have gained more attention of researchers in identifying, screening and characterising antioxidants to replace the synthetic ones. Numerous studies have reported that polyphenols and their rich extract can reduce oxidative stress associated with the chronic inflammatory diseases [38–40]. In the present study, *in vitro* antioxidant activity was analysed by DPPH free radical scavenging assay and reducing power capacity assay. AFMV exhibited a significant (*P<0.01*) correlation between extract and ascorbic acid for the percentage of inhibition in scavenging abilities. The IC_50_ values of AFMV indicated that it has promising protein donating ability and might serve as a free radical inhibitor. The reducing power capacity of AFMV extract was evaluated by using Fe^3+^ to Fe^2+^ reduction assay. The results of the mentioned assay reveal significant reduction (*P<0.05*), where the yellow colour of test solution shifts to different shades of blue and green colour, based on the reduction capabilities of sample [41]. The results depicted that AFMV can donate electrons which can react with free radical and energetically impede radical chain reaction.

The thrombolytic activity of the AFMV was evaluated and compared with positive and negative control, respectively. Available marketed thrombolytic agents demonstrate their action by blocking thrombus formation in the blood vessel, thereby plasminogen converts into plasmin, resulting in clear tracery of cross-linked fibrin. However, these agents also show severe adverse effects are major clinical drawback also linked to many limitations [1]. Phytochemicals from several ethnomedicinal sources likely, vegetables and fruits are reported to have anticoagulant, antiplatelet and fibrinolytic potential and hence able to mitigate instance of coronary complications and stroke. AFMV was also found to provide significant thrombolytic activity in this study. This inhibition of clot formation could be attributed to various phytochemicals in it.

Denaturation of intra-cellular protein is a well-known reason for rheumatoid arthritis and other inflammatory diseases. The inhibitory activity against protein denaturation is one of the most common methods to investigate the anti-arthritic activity of the plant extracts. Protein denaturation is associated with heat, superficial anxiety and chemical vulnerability which helps to generate auto antigens-associated type-III hypersensitivity reaction in certain arthritic diseases [42]. The denaturation process possibly involves modifying the electrostatic, hydrogen, hydrophobic and disulphide linkages [43]. The ability of an agent to prevent protein denaturation suggests the potential of being an anti-arthritic agent. The present experimental findings showed that AFMV exhibited a significant and dose-dependent inhibitory percentage that was parallel to the reference drug. Secondary metabolites include triterpenoids, phenols, tannins and flavonoids might be responsible for this anti-arthritic activity. Research also suggests that flavonoids have the specific potential to inhibit protein denaturation [44,45].

Pain is one of the most pervasive problems and results due to multiple nociceptive and inflammatory mediators activation such as prostaglandin, bradykinin, histamine, tumour necrosis factor-α, IL-1β, IL-8, which ultimately helps to form edema, inflammation and invasion of leukocytes [46,47]. Various available marketed anti-inflammatory and anti-nociceptive drugs include non-steroidal anti-inflammatory drugs, opioids and glucocorticoids are widely used for their significant action at peripheral sites, but these drugs show adverse effect and inefficient sometimes; therefore, investigation of new drugs is needed to overcome those limitations. AFMV extract in the current was investigated against the central and peripheral acting analgesia model by acetic acid-inclined writhing test and formalin-inclined paw licking assay, respectively. Although the acetic acid-induced test showed good sensitivity, sometimes this test showed poor specificity. Research has been suggested that the intraperitoneal administration of acetic acid injection into the peritoneal cavity leads to an elevated level of COX-2 and lipoxygenase. In the present study, AFMV extract significantly decreased acetic acid-induced writhing in a dose–response manner, and these results were found parallel with the reference drug. Therefore, present outcomes in acetic acid-induced mice model suggested that the anti-inflammatory activity of AFMV might be linked partly with the reluctance of COX-2, lipoxygenase and other inflammatory mediators in peripheral tissue hence hindering with the signal transduction pathways in main afferent nociceptors. The formalin-induced paw licking test was also employed in the present study to confirm the action of AFMV against central and peripheral anti-nociceptive pain. This is a biphasic model characterised by two distinct phases, neurogenic pain (0–5 min) and inflammatory pain (15–30 min), respectively [48]. After administration of formalin, early phase indicates acute response caused by the direct effect of formalin on the sensory C-fibers, while the late phase reveals delayed response by the release of inflammatory mediators [49]. In this present study, oral administration of AFMV significantly (*P<0.001*) suppressed pain sensation in both phases as confirmed by reduced licking behaviour, but the impact in the late phase was more prominent. Phytochemicals in AFMV such as alkaloids and flavonoids might be responsible for its inhibitory activity against pain in the experimental model.

## Conclusion

The study evaluated antioxidant and anti-arthritic effects of AFMV through *in vitro* models. The study also examined associated biological effects such as thrombolytic action and *in vivo* anti-nociceptive activity. Based on the data, AFMV could be a potential source of antioxidant, anti-arthritic, thrombolytic and anti-nociceptive effects. These effects possibly attributed to secondary metabolites (e.g. alkaloids, flavonoids, tannins and triterpenoids) present in the plant. However, AFMV needs to be further analysed by various techniques such as high-performance liquid chromatography, nuclear magnetic resonance and mass spectrometry for the identification of its major isolates to elucidate the underlying mechanism responsible for the activities.

## Supplementary Material

Supplementary Table S1

## Data Availability

All data are included in the manuscript

## Open Access

Open access for this article was enabled by the participation of University of Melbourne in an all-inclusive *Read and Publish* pilot with Portland Press and the Biochemical Society under a transformative agreement with CAUL.

## Abbreviations

AFMV: aqueous fraction of *Merremia vitifolia* leaves
COX-2: cyclooxygenase-2
DPPH: 2,2-diphenyl-1-picrylhydrazyl
RA: rheumatoid arthritis
ROS: reactive oxygen species
SK: streptokinase
