# Supplementary material for: Pharmacological insights into Merremia vitifolia (Burm.f.) Hallier f. leaf for its antioxidant, thrombolytic, anti-arthritic and anti-nociceptive potential
Source: Biosci Rep. 2021 Jan 7;41(1):BSR20203022. doi: 10.1042/BSR20203022 (PMC7791546; doi:10.1042/BSR20203022)
Supplement: Supplementary Table S1 [file BSR-2020-3022_supp.pdf]

Table S1. Phytochemical Screening of *Merremiavitifolia*

Group Test	Name of the test	Observation
Alkaloid	Meyers Test	++
	Wagners Test	++
Carbohydrate	Molish Test	+
	Benedict Test	+
Flavonoids	Hydrochloric acid Test	+++
Saponins	Foam Test	_
Tannins	Potassium dichromate Test	+
Glycoside	Acetic acid test	_
Protein	BSA Test	+
Phenolic Compounds	Ferric chloride test	++
Triterpenoids	Acetic anhydride test	++

Here, '+' Present, '++' Rapidly present, '-' Absent
